# Atrial Mass in Pregnancy: A Rare Case of Intracardiac Ectopic Liver

**DOI:** 10.1016/j.atssr.2025.02.006

**Published:** 2025-03-04

**Authors:** Armita Kabirpour, J. Ross Wheeler, Jerry Saunders, Sara Edwards, Solomon Bienstock, Mary B. Beasley, Robin Varghese

**Affiliations:** 1 Department of Cardiovascular Surgery, Mount Sinai Health System, New York, New York; 2 Department of Pathology, Molecular and Cell-Based Medicine, Mount Sinai Health System, New York, New York; 3 Department of Obstetrics and Gynecology, Mount Sinai Health System, New York, New York; 4 Zena and Michael A. Wiener Cardiovascular Institute, Mount Sinai Health System, New York, New York

## Abstract

We report a case of a 41-year-old female who was found to have a 2.7-cm right atrial mass during the third trimester of her pregnancy. She underwent successful surgical resection 4 months after an uneventful delivery. Pathologic examination revealed an ectopic liver, a remarkably rare finding in this anatomic location. The potential for malignant transformation, embolization, obstruction and the need for histologic diagnosis necessitated surgical removal of the intracardiac ectopic liver. We discuss the role of cardiovascular imaging and considerations for surgical planning. In addition, we review the hormonal alterations during pregnancy that may promote growth of hepatic tissue.

The presence of hepatic tissue outside of the native liver is referred to as an ectopic liver.^1^ There are only a handful of reported cases of intracardiac ectopic liver, and it is often initially misdiagnosed as a myxoma or thrombus.^2^, ^3^, ^4^, ^5^ The microscopic features of ectopic hepatic tissue are similar to normal liver; however, it lacks the complete functional architecture and is prone to malignant transformation.^6^ Surgical removal is indicated, given the potential for carcinogenesis, obstruction, and embolization. The pathogenesis of ectopic liver is unclear. Trocciola and colleagues^4^ offer several hypotheses on its cause, including embryologic formation of a second liver bud, cellular migration from the original liver bud, and incomplete atrophy of developing liver lobes.

A 41-year-old African American woman G3P2002 presented with sinus tachycardia during her third trimester and was found to have a 2.7-cm well-circumscribed mass in her right atrium (RA) on transthoracic echocardiography (TTE; Figure 1A). Use of a percutaneous suction device was not an option, given the firm attachment of the mass to the atrial wall.

**Figure 1:**
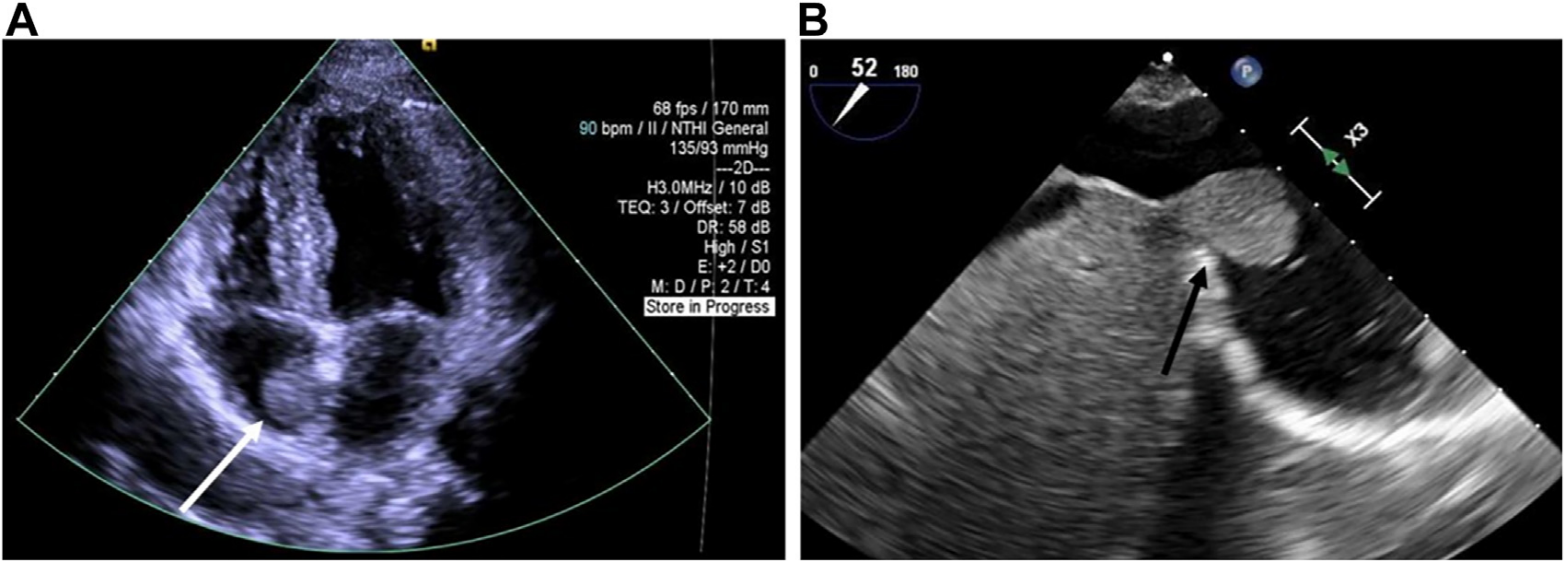
(A) Mobile mass in the right atrium measuring 2.7 × 2.2 cm on transthoracic echocardiogram (arrow). (B) On intraoperative transesophageal echocardiogram, the mass was better visualized close to the liver (arrow).

As the mass was on the right side, stable in size on repeat TTE, without obstruction or valvular dysfunction, we observed her until the 37th week of gestation and allowed a 4-month recovery period following an unremarkable cesarean delivery before surgical resection. Intraoperative transesophageal echocardiography (TEE) showed the mass at the inferior cavoatrial junction (Figure 1B). The right femoral vein was cannulated percutaneously with the tip positioned in the intrahepatic inferior vena cava (IVC), given the proximity of the mass to the inferior cavoatrial junction. We proceeded with central cannulation of the ascending aorta and superior vena cava by our standard-approach small-incision median sternotomy. Antegrade cardioplegia was used to arrest the heart. Right atriotomy revealed a well-circumscribed mass attached by a stalk at the inferior cavoatrial junction without tricuspid valvular involvement. The mass was resected with a small endocardial margin. The resected reddish-brown lobulated specimen measuring 2.8 × 1.8 × 1.7 cm was submitted for pathologic examination (Figure 2). The cavoatrial wall where the mass was resected was then cryoablated and oversewn. Microscopic examination revealed typical features of hepatic parenchyma with normal lobular configuration including portal triads and central veins. Foci of chronic inflammation were present within some of the portal triads. The hepatocytes were arranged in a single layer and lacked atypical features (Figure 3). The patient’s postoperative recovery was uneventful.

**Figure 2:**
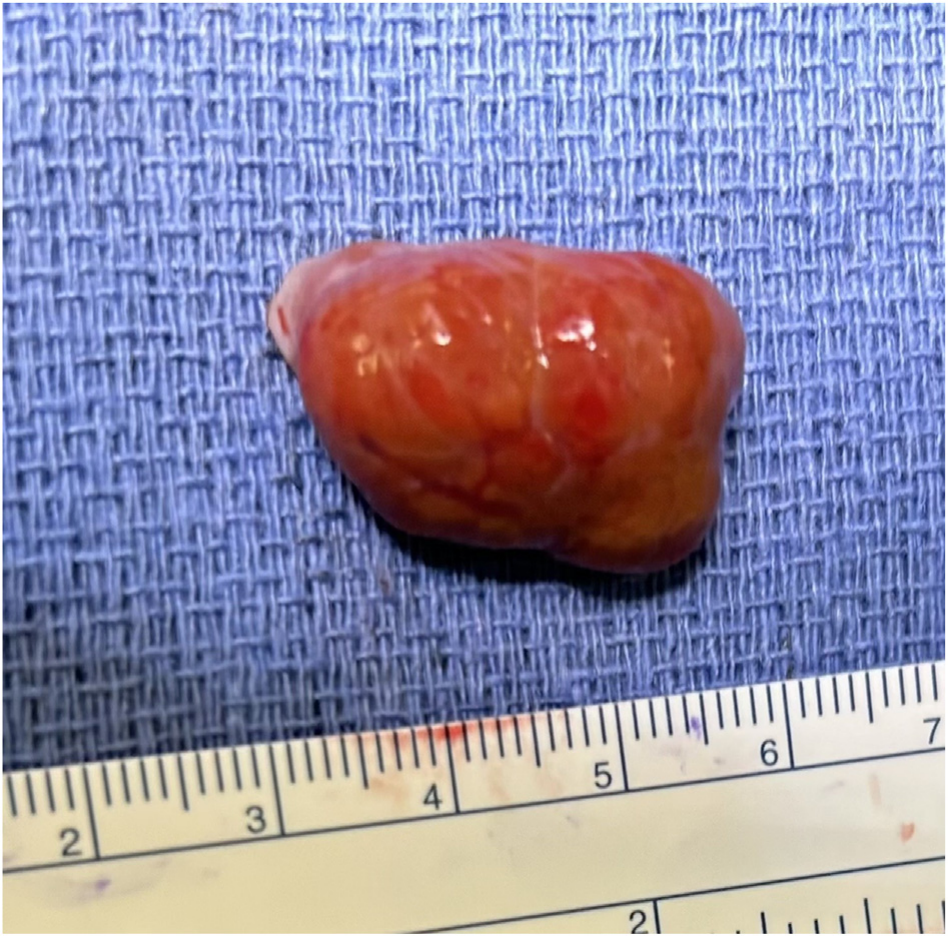
A reddish-brown lobulated soft tissue specimen measuring 2.8 × 1.8 × 1.7 cm was resected from right atrium along with endocardium.

**Figure 3:**
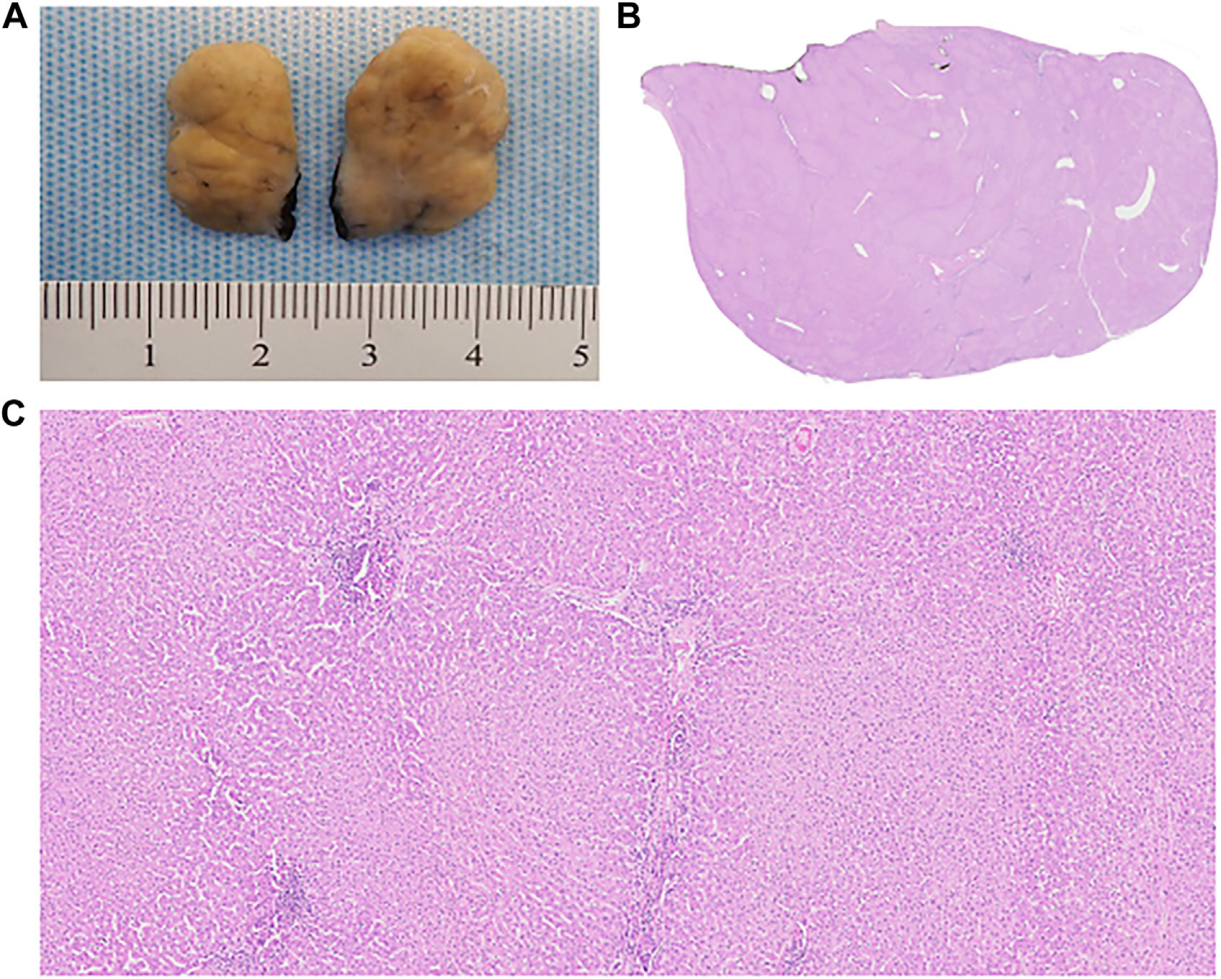
(A) Gross specimen bisected with a firm, tan, lobulated appearance lacking myxoid features. (B) Low-power image demonstrates that the entire mass is composed of hepatic tissue with no areas showing features of myxoma (magnification ×5). (C) At higher power, the tissue shows typical features of normal hepatic parenchyma with a normal lobular configuration including portal triads and central veins. Foci of chronic inflammation are present within some portal triads. The hepatocytes are arranged in a single layer and lack atypical features (magnification ×50).

## Comment

Although rare, an ectopic liver should be considered in the differential diagnosis for right-sided intracardiac mass. Initial workup includes TTE to localize and measure the size of the mass and to identify additional structural abnormalities. TEE provides a more precise view of the involvement of surrounding structures. Cardiac magnetic resonance (CMR) imaging may aid in better delineating tissue characteristics. TEE or CMR would allow one to determine whether the mass is thrombus or tumor with firm intracardiac attachment to guide the choice for the optimal treatment modality, for example, catheter thrombectomy vs surgical resection. Both TEE and CMR could also be used for operative planning to better characterize the mass and its relationship to the intrahepatic IVC, interatrial septum, tricuspid valve, or coronary sinus. For example, if the mass location compromises drainage on cardiopulmonary bypass, alternative cannulation strategies or hypothermic circulatory arrest may be necessary.^7^ Depending on the extent of IVC involvement and need for reconstruction, a hepatobiliary surgeon may be necessary.^5^

Several cases of intracardiac ectopic liver were reported in women of childbearing age with obesity. Doshi and coworkers^2^ described a 46-year-old woman with morbid obesity, hypertension, and hyperlipidemia presenting with atrial fibrillation who was found to have a right atrial mass that was subsequently diagnosed as an ectopic liver following resection. Izzo and coworkers^3^ described a 43-year-old woman with prediabetes presenting with syncope, found to have an ectopic liver at the RA-IVC junction. Trocciola and colleagues^4^ presented a case of an obese 42-year-old woman presenting with palpitations who had an ectopic liver at the RA-IVC junction. Giritharan and colleagues^5^ described a 30-year-old woman with endometriosis, obesity, and history of cholecystectomy presenting with chest pain and found to have a right atrial ectopic liver. These reported cases of ectopic intracardiac liver all appeared to occur in female patients with metabolic derangements, which have led prior authors to speculate about a potential hormonal contribution in the growth of ectopic intracardiac liver.

With this mass presenting during pregnancy, this led us to inquire whether the hormonal changes of pregnancy influence the growth of heterotopic liver tissue. It is known that estrogen and progesterone levels rise during pregnancy with a substantial upregulation during the third trimester. These hormones are known to increase hepatic synthetic function during pregnancy. In addition, prolonged exposure to estrogen and androgens has been shown to induce regenerative and dysplastic hepatocellular changes.^8^ Although data are limited, our case report again suggests that the hormonal changes of pregnancy may play a role in the development of ectopic liver.

Although intracardiac ectopic liver tissue is extremely rare, it should be considered in the differential diagnosis of RA masses. Initial workup should begin with TTE, followed by TEE or CMR to better characterize the mass and to determine the extent of anatomic involvement to guide the optimal strategy for intervention. Ectopic liver is usually diagnosed on microscopic examination after resection. Surgical resection is also indicated, given the potential for malignant transformation, obstruction, and embolization.
